# New Researches on Hæmatology

**Published:** 1853-04

**Authors:** A. Becquerel, A. Bodier


From the Medicale de Paris.

*New Researches on Hæmatology.*—By MM. A. BECQUEREL and A. BODIER.

[The following are the conclusions of a long series of observations upon morbid changes in the blood, which have been recently communicated to the Academie des sciences, and reported in the above named journal.]

1st. In the majority of chronic maladies, and in various other unhealthy conditions, there is some increase or diminution in the normal proportions of the three principal elements of the blood—the globules, the fibrine, and the albumen, and this in a single, double, or triple order.

2d. The *globules* diminish in number in the course of most protracted chronic disorders, and especially in organic affections of the heart, the chronic form of Bright's disease, chlorosis, march-cachexy, hemorrhages of various kinds, excessive blood-lettings, the last stages of tubercular disease, and the cancerous diathesis. The globules disappear equally in those who are sunk in the depths of poverty, and exposed to the conjoined evil of bad and insufficient food, and of dark, damp, and ill-ventilated dwellings.

3d. The *albumen* of the serum of the blood diminishes, among other instances, in Bright's disease, the march-cachexy, advanced heart disease, great symptomatic anæmia, and in the state of ill health induced by cancer and by poverty.

4th. The proportion of *fibrine* is unaffected, or slightly augmented, in acute scorbutus; but diminished in the chronic malady, especially in that symptomatic form which often complicates permanent heart disease.

5th. In all the cases already mentioned, the quantity of *water* contained in the blood is considerably augmented.

6th. The more prominent signs of a diminution in the number of globules are the following:—discoloration of the skin, palpitation, dyspnœa, *bruit de soufflet* at the base of the heart during its first sound, an *intermittent bruit de soufflet* in the carotids, and a *continuous* one in the jugulars.

7th. A rapid, though slight, *diminution in the quantity of albumen*, is marked by acute dropsy. A more gradual diminution is followed by the same symptom, but in this instance the loss must have been much more considerable than in the former one. Dropsy, in fact, is the characteristic sign of a blood deprived of its natural amount of albumen.

8th. A diminution in the normal proportions of *fibrine* is followed by hemorrhage of one kind or another—mucus or cutaneous.

9th. In the *anæmia*, which is symptomatic of copious hemorrhage, starvation, or exhausting discharges, the blood is less dense and more watery than natural, the globules are diminished in number, and the albumen and fibrine unaffected, or the former slightly wanting.

10th. In *chlorosis*, which is an affection distinct from anæmia, the blood may be unchanged. If it is changed, it has fewer globules and more water, and a natural or somewhat augmented proportion of fibrine and albumen.

11th. In *acute Bright's disease*, the globules and fibrine remain unaltered, and the albumen is wasted. In the *chronic* affection, the globules, as well as the albumen, are deficient, and not unfrequently the fibrine also, though to a less extent than the others.

12th. Most of the idiopathic *dropsies* are due to a want in the normal quantity of the albumen of the blood.

13th. In *fatal diseases of the heart*, the blood progressively becomes more and more impoverished in its three elements of fibrine, albumen, and globules, while at the same time it is rendered much more watery.

14th. In *acute scorbutus*, the blood presents no appreciable alteration. In the *chronic* affection, the blood is notably deficient in fibrine, while the globules are, sometimes at least, considerably increased.

15th. These facts should exercise a great influence in practice, as we possess the means of acting upon the element which may be wanting or changed. A tonic plan of treatment will be required when each of the three elements is deficient, combined with quinine, steel, or acids, according as the deficiency may be in the albumen, globules, or fibrine—one reason of the indication of acids in the latter case being the presence of free soda in the blood when the fibrine is deficient.

